# Fronto-orbital advancement and reconstruction using reverse frontal bone graft without the use of orbital bar: a technical note

**DOI:** 10.1007/s00381-020-04583-w

**Published:** 2020-03-26

**Authors:** James M. W. Robins, Asim J. Sheikh, Dmitri Shastin, Moritz W. J. Schramm, Paula Carter, John L. Russell, Mark Liddington, Paul D. Chumas

**Affiliations:** 1 Department of Neurosurgery, Leeds General Infirmary, G Floor, Jubilee Building, Leeds, LS1 3EX UK; 2 Department of Maxillofacial Surgery, Leeds General Infirmary, Great George St, Leeds, LS1 3EX UK; 3 Department of Plastic and Reconstructive Surgery, Leeds General Infirmary, Great George St, Leeds, LS1 3EX UK

**Keywords:** Craniofacial, Synostosis, Metopic, Coronal

## Abstract

**Introduction:**

We describe our technique of using reverse frontal bone graft for FOAR for patients with metopic or coronal synostosis and present our complications using the Leeds classification system for complications in craniosynostosis surgery.

**Methods:**

Since April 2015, seventeen patients have been operated using this technique. We perform a frontal bone graft that is then reversed, and supraorbital margins are drilled out. The orbital bar is then removed and drilled down to make bone dust and on-lay bone grafts which are then used to fill gaps on exposed dura and fill in around the temporal region.

**Results:**

All 17 patients who underwent this technique have good cosmetic results. We report 5 (29%) complications and 8 (47%) blood transfusions (7 exposures, 1 cell salvage).

## Introduction

Coronal and metopic synostosis pose a specific challenge for surgical treatment having evolved from simple suturectomy to fronto-orbital advancement and reconstruction (FOAR) [1, 2]. Multiple FOAR techniques are described using templates for frontal bone graft [2], wire fixation or rigid metallic fixation or the use of resorbable plates. Orbital bar modifications include leaving intact, advancing the bar forward or, as demonstrated here, removing it altogether [3].

Remodelling techniques for metopic correction are recently described including the shell technique [4], cathedral dome procedure [5] and Lille’s frontal reshaping and rotation of the superior and lateral orbital rim [6]. Absorbable plates offer an alternative to rigid fixation but carry higher complications [7, 8]. A recent technique of orbital buttress offers an alternative to screws and plates altogether [9]. None of these techniques however addresses the problem of thinning in the bitemporal regions.

Our technique for FOAR has evolved over the years from the standard Marchac template technique, to thinning down the inner table of the orbital bar so as to be able to better reshape it [10], through to our present procedure—where the orbital bar is removed but only used for bone dust and on lay grafts. The bone dust and grafts are used to fill the temporal area to avoid future thinning and to fill gaps at exposed dura. This aids bony fusion and provides a favourable cosmetic outcome. We describe the technique, outcomes and complications of this method in a single institution.

## Methods

### Patient selection

All paediatric patients presenting with non-syndromic metopic or coronal synostosis since April 2015 underwent this technique in a single institution.

### Surgical technique

#### Positioning, preparation and incision

Patients are supine with head on horseshoe rest. Pressure areas are protected and the corneas covered with chloramphenicol antibiotic cream. The skin is prepared with aqueous iodine-based solution.

**Fig. 1:**
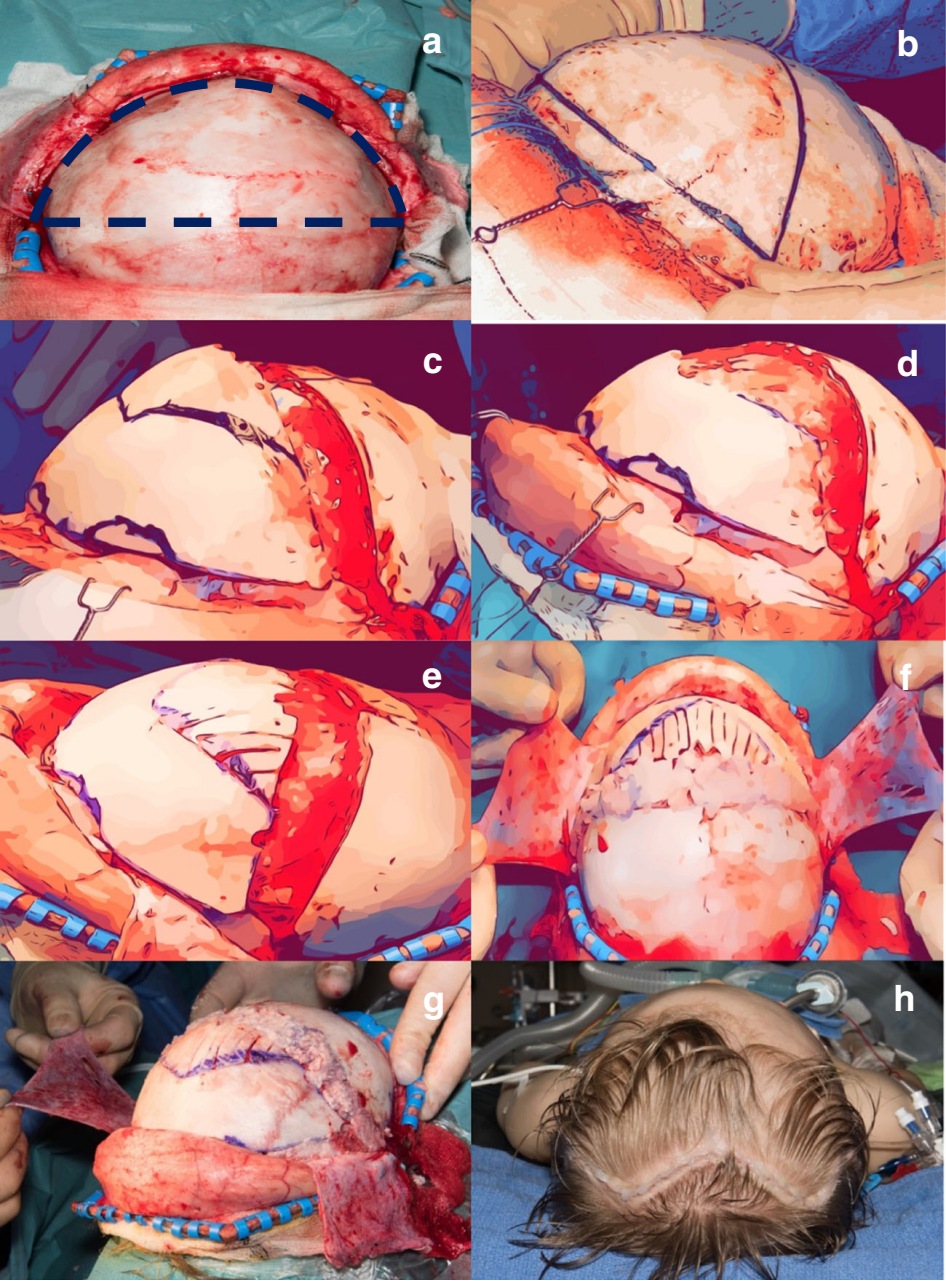
Exposure and marking **a**, bone flap and orbital bar marking **b**, markings for next cut **c**, new frontal graft in place **d**, frontal graft with barrel staved part **e**, bone dust from removed orbital bar used to cover exposed dura **f**, bone removed from orbital bar used to fill biparietal gaps **g**, immediate post-op appearances **h**

A zigzag bicoronal incision is fashioned [Leach 2004] and flaps dissected to expose orbital rims anteriorly (Fig. 1a). The pericranium is divided in the midline and taken down bilaterally with the temporalis muscle.

### Frontal flap marking and removal of orbital bar

Frontal bone flap is marked and removed. The orbital bar is then removed using standard technique with the cuts along the anterior fossa floor being as close to the orbital rim as possible (Fig. 1b, c).

### Bone flap reversal

Frontal bone flap is then reversed, and new orbital rims are marked and fashioned as shown. In metopic cases, the new construct is not advanced, but in coronal cases, it is advanced as far as the soft tissue envelope will allow, typically 1.5–2.0 cm. As this advancement is symmetrical, in cases of unicoronal synostosis, the advancement does not appear as an “over advancement” as seen in other techniques but will be significantly more advanced on the affected side. The advancement is maintained by resorbable LactoSorb plates and lag screws placed in the temporal region bilaterally and absorbable sutures to nasion (Fig. 1d).

### Reconstruction

The remaining bone strip is barrel staved to be placed in the gap (Fig. 1e). Bone fragments from removed orbital rim are sited temporally to prevent temporal thinning (Fig. 1f). Remaining orbital bar is drilled to bone dust and covers any remaining exposed dura (Fig. 1g). The pericranium is tacked together to help hold the construct in place.

Layered wound closure is with absorbable sutures for galea and monofilament subcuticular for skin (Fig. 1h).

## Results

### Demographics

Between April 2015 and March 2019, we performed this procedure in 17 non-syndromic patients (9 female, 8 male; age range, 12–33 months; mean age, 19.2 months). There were eight unicoronal and nine metopic synostosis. Follow-up ranged from 1 to 34 months (median 16.2 months) and length of stay ranged 2–7 days (mean 4 days).

### Cosmetic outcomes

**Fig. 2:**
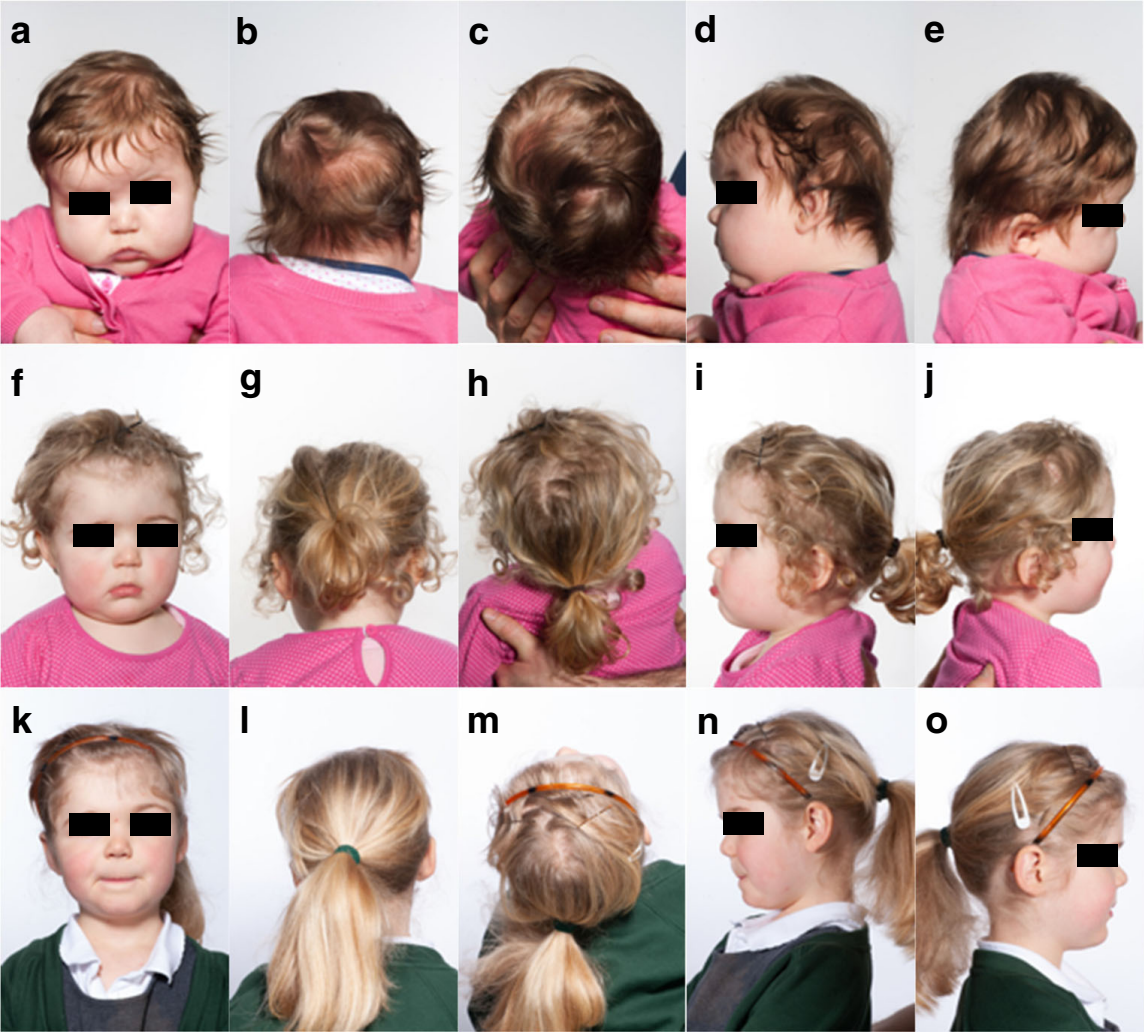
Pre- (**a**–**b**) and post-op appearances at 18 (**f**–**j**) and 34 months (**k**–**o**) for unicoronal synostosis

All patients had pre- and postoperative photographs taken for comparison, and all had satisfactory cosmetic outcomes (Fig. 2). Two patients had subtle forehead recession and one patient has a slightly uneven vertex at follow-up; however, none required reoperation, and parental satisfaction was confirmed. Ophthalmological follow up did not demonstrate pulsating exophthalmos in any patients at this length of follow up.

### Radiological outcomes

Whilst we used to obtain standard skull X-rays as baseline postoperatively, we have moved to specially protocolled low dose CT head with 3D reconstruction as routine in our institution, and this confirmed good radiological outcomes in all patients.

### Blood loss and transfusion exposure

Seven patients (41%) underwent eight blood transfusions from seven blood bags; of which, three were intraoperative (one cell salvage) and five postoperative (one patient had intraoperative and postoperative transfusion). Five patients (29%) received a sodium feredetate only, whilst one received sodium feredetate and intraoperative transfusion and, one combined with a postoperative transfusion. In the previous results in our institution [11], 31 patients (78%) underwent blood transfusion (2 cells salvage) of which 21 were intraoperative (52.5%) and 11 postoperative (27.5%). Again, one patient had intra- and postoperative transfusion. There was no difference in volumes transfused intra- and postoperatively (p 0.094, p 0.467) in ml/kg between studies; however, there was almost a significant decrease (p 0.0551) in total blood volume transfused in this new technique compared with the previous technique used in our unit (Table 1).

**Table 1:** Comparison of mean surgical time and transfusion volume between new and previous FOAR technique in our institution

	No of patients	Mean surgical time/h	*P* value	Blood transfusion intra-op			Blood transfusion post-op		
				No.	Mean volume	*P* value	No.	Mean volume	*P* value
Robins et al	17	3:33	0.32	3	11.84 ml/kg	0.094	5	13.67 ml/kg	0.467
Shastin et al	40	3:23		21	17.50 ml/kg		11	15.08 ml/kg	

### Surgical time

Theatre time from incision to closure ranged from 3:01 to 4:31 h (mean 3:33 h, median 3:30 h). Note no significant increase in time compared with the previous technique used in our institution (Table 1).

### Complications

There were no mortalities; however, five patients (29%) experienced complications.

One patient experienced a dural tear, repaired intraoperatively, and then subsequently represented with a culture-positive wound infection (*Salmonella* sp.). This required four washouts and IV antibiotics. One patient suffered some blood-stained vomit managed conservatively with no increased length of stay. Three patients had slight forehead recession or an uneven vertex; however, none required reoperation. Complications along with their Leeds classification of complications in craniosynostosis surgery are demonstrated in Table 2 [11].

**Table 2:** Demonstration of complications and classification using Leeds classification of complications in craniosynostosis compared with Oxford classification [11]

Complication	No. of patients	Leeds classification	Oxford classification
Wound infection	1	3C—Complications requiring readmission. Surgical 30 ≤ days since discharge	3—Reoperation but no long-term sequelae
Blood-stained vomit	1	1A—Inpatient complication with normal LOS	1—No delay in discharge, reoperation or long-term sequelae
Slight forehead recession	2	2B—Outpatient complications not requiring readmission	1—No delay in discharge, reoperation or long-term sequelae
Uneven bumpy vertex	1	2B—Outpatient complications not requiring readmission	1—No delay in discharge, reoperation or long-term sequelae

## Discussion

Surgical treatment for craniosynostosis continues to evolve. Original techniques involved strip craniectomy; however, this did not address cranio-orbital deformities [12]. Simple suturectomy does not address orbitocranial deformity or associated risk. Subsequently fronto-orbital advancement was shown to be a safe technique [2] and was refined to correct skull base deformity and improve cosmetic outcomes [13].

Thornett et al. presented a FOAR series without removal of orbital bar in 2016 [14]. We present a similar technique but with orbital bar removal. We remove the frontal bone via bifrontal craniotomy and reverse it. New orbital rims are fashioned in this graft and removed orbital bar fills bitemporal gaps. This aims to prevent graft torsion and future biparietal thinning. The remaining bone is drilled providing bone dust to cover exposed dural surfaces and promote fusion. In our experience, this results in good cosmetic outcomes—including in the temporal region, frequently a problem in other techniques, especially metopic synostosis. We found no persistent temporal “thinning” in our series; however, this may only be evident at prolonged follow-up, and thus this limits our conclusions. We did notice two examples of mild forehead recession despite advancing the orbital rim as far as soft tissue envelope allowed. Again, we accept that follow-up is still limited—although the case of unicoronal synostosis in Fig. 2 shows a good result at 34 months. It may be that the risk of late recession is reduced by the use of the bone originating from a different region. Likewise, it is of note that transposition of different cells from orbital bar to another skull region does not result in mucocele formation, provided grafted bone does not contain frontal sinus precursor cells [15].

We also present a low rate of complications in this initial small series and demonstrate these using a craniosynostosis classification system [11]. We show no difference in volume of intra- and post-op blood transfusion compared with the previous work; however, there is almost a significant decrease in total blood volume transfused (intra- and post-op combined) [11] suggesting a possible impact from newly using intraoperative tranexamic acid in all patients in this series. This is likely to be significant in a larger case series. Of note is that there was no difference in operative time using this new technique. Length of stay was on average 4 days, comparable with previous findings.

## Conclusion

We demonstrate a novel technique of FOAR for metopic and coronal synostosis. Our low complication rate shows that it is safe to remove orbital bar, utilising it as bone dust to fill temporal gaps. This achieves forehead flattening, minimal frontal bone flap torqueing and appropriate advancement with prevention of parietal thinning at this limited length of follow-up.
